# The Influence of Comminuting Methods on the Structure, Morphology, and Calcium Release of Chicken Bones

**DOI:** 10.3389/fnut.2022.910435

**Published:** 2022-05-31

**Authors:** Ying Wang, Tao Feng, Qiang Xia, Changyu Zhou, Jinxuan Cao

**Affiliations:** ^1^School of Food and Health, Beijing Technology and Business University, Beijing, China; ^2^State-Owned Assets and Laboratory Management Office, Anhui Polytechnic University, Anhui, China; ^3^College of Food and Pharmaceutical Sciences, Ningbo University, Ningbo, China

**Keywords:** calcium supplement, chicken bone, comminuting methods, calcium release, particle size, structure

## Abstract

This work aimed at assessing the influence of comminuting methods, including colloid mill, planetary ball mill and dynamic high-pressure microfluidization on the chemical composition, particle properties, morphology and calcium release of chicken bone. The results showed that planetary ball mill and dynamic high-pressure microfluidization could reduce the particle size of bone powder, and the particle size of sample treated by dynamic high-pressure microfluidization reached 446 nm. Chicken bone particles were negatively charged, and the absolute value of zeta potential was significantly reduced after milling treatments. Furthermore, X-ray diffraction and Fourier-transform infrared spectroscopy (FTIR) analysis indicated that the planetary ball mill and dynamic high-pressure microfluidization processes presented no significant effect on the internal chemical structure of bone particles. Compared with the other groups, samples treated by dynamic high-pressure microfluidization released more calcium ions, which was related to the significant effects on surface calcium composition and reducing particle size. Therefore, dynamic high-pressure microfluidization has a great potential in the processing of bone-derived products, particularly for the design and development of bone-derived product with high calcium bioaccessibility.

## Introduction

Calcium is essential for the development of human bones, tooth remineralization, and a variety of other physiological processes; in particular, adequate calcium uptake contributes to the prevention of osteoporosis in the elderly population and menopausal women (1). As an essential element in the human body, calcium can only be obtained from dietary sources. However, the data from the evaluation on the nutrient intake of Chinese adults demonstrated that the mean intake of calcium from food sources was only 391 mg/day, far below the recommended daily intake for Chinese residents. Therefore, fortification of calcium ingestion is of great importance, especially in the development of calcium supplements with high absorption of bioavailable calcium ions (2). The major problems with commercial calcium supplements lie in unsatisfactory absorption efficiency, high cost, and possible adverse effects, such as naupathia and constipation; hence, approaches aimed at enhancing the solubility and absorption of calcium have been explored over the last several decades (2).

Animal bones, which are rich in proteins, minerals, and other nutrients, can be used as a natural, low-cost source of calcium with good bioavailability and biocompatibility (3). Extensive studies have focused on the feasibility of producing animal bone powder, particularly fishbone, as food supplements to enhance product quality (4). Li et al. (5) demonstrated that tuna bone powder significantly contributes to the alleviation of glucocorticoid-induced osteoporosis by coregulating signal pathways. It has been found that adding silver carp bone powder to fish sausage improves the calcium supplement properties (6).

Driven by the growing demand for poultry meat, the production of broiler in China has been expanding significantly over the past few decades, reaching approximately 14.58 million tons in 2018 (7). As the by-products of chicken slaughtering plants, most of the chicken bones were directly discarded or mainly processed into low-value animal feed ingredients, leading to environmental contamination or resource waste. Previous researchers have noticed that not only are chicken bones rich in calcium, but they also have a ratio of calcium to phosphorus of 2:1 (8), meeting the proposed compositional requirements for body absorption (9). Therefore, chicken bones have great potential for deep processing to improve their utilization in the food industry. Chicken bones can be expected to be utilized as an edible powder for calcium supplements.

As a common technology for the manufacture of nanomaterials, milling processes have been demonstrated to influence the physicochemical features of animal bone powder significantly, such as bone particle size, chemical forms of calcium, and matrix structure, all of which further determine the release of calcium (10). Among the present processing methods, the most widely used physical comminuting preparation methods for bone powder include colloid mill, planetary ball mill, and dynamic high-pressure microfluidization. Liu et al. (11) obtained maitake mushroom powder using the colloid mill pretreatment method. Wu et al. (12) and Li et al. (13) prepared micron-fish bone and nano-rabbit bone particles by superfine grinding and planetary ball milling. Chen et al. (14) utilized the dynamic high-pressure microfluidization process to change the properties of polysaccharides, including particle size, water holding capacity, and apparent viscosity. Overall, the effects of comminuting treatments on the characteristics of powder products vary depending on the grinding method and raw materials (10). However, there is little information about the comparative effects of different comminuting treatments on the physicochemical properties of chicken bone meal.

Our work aimed to compare the functions of colloid milling, ball milling, and dynamic high-pressure microfluidization treatments for the preparation of chicken bone powders, as well as their applicability for producing low-cost, high-performance calcium supplements. Correspondingly, the physiochemical properties of chicken bone powders obtained from different comminuting treatments were investigated, including their morphology, structure, and calcium release. These results should provide the theoretical basis for the selection of comminuting methods while simultaneously enhancing the utilization and the added value of chicken bone.

## Materials and Methods

### Materials

A frozen chicken skeleton (45 days old, Rose 308) was purchased from New Hope Liuhe Co., Ltd. (Zhejiang, China). The chicken bone was thawed to remove the remnants of meat and other substances and then washed several times. Clean bones were stored at −18°C before use. All chemical reagents, including papain (2,000 U/mg protein, Sangon Biotech Co., Ltd., Shanghai, China), were of analytical grade, and deionized (DI) water was used in all the experiments.

### Preparation of Bone Meal by Different Milling Approaches

#### Colloid Milling

Frozen chicken bones were thawed at room temperature before being uniformly chopped into pieces. Since some specific groups in protein and peptides could affect the release of calcium through binding with calcium, organic components of chicken bone were removed before milling to avoid interference. The pieces of chicken bone were boiled down (at 121°C for 40 min) in an automatic high-pressure steam sterilizer (LDZF-50L-II, Shanghai Shenan Medical Instrument Factory, Shanghai, China), which resulted in the partial dissociation of collagen from the bone. After removing the floating fat and liquid, papain was used to further eliminate tightly bound proteins from the chicken bones using the method described by Dhakal et al. (15) with slight modifications. Bones were submerged in phosphate buffer (0.1 M, pH 7) at a ratio of 1:3 (w/v), then hydrolyzed at 50°C by papain (the enzyme addition was 5,000 Ug^–1^) for 4 h and inactivated by boiling for 10 min. Next, chicken bones were coarsely crushed twice using a meat mincer (BS216B, Hengshui Yixuan Kitchen Equipment Trading Co., Ltd., Hebei, China) through 3-mm diameter pores in a sieve plate. The bone mince was mixed with ice water (approximately a 1:1.5 ratio) and grounded using a colloid mill (JMS-130C-1V, Langfang City Langtong Machinery Co., Ltd., Hebei, China) at 2,940 rpm cyclically for three times, during which the space between the disk was set to 4 μm. Finally, the mixture was spray dried (YC-018, Shanghai Yacheng Instrument Equipment Co., Ltd., Shanghai, China). The obtained chicken bone meal was passed through a 200-mesh standard sieve (Zhejiang Shangyu Huafeng Hardware Instrument Co., Ltd., Zhejiang, China) prior to subsequent processing. This chicken bone powder was labeled as colloid milling bone meal (CBM).

#### Ball Milling Treatment

Ball milling of bone particles was performed in a manner similar to that described by Li et al. (13) with minor modifications. CBM was then processed in the planetary ball mill (QM-3SP2, Nanjing Nanda Instrument Co., Ltd., Nanjing, China) under the following suitable operating parameters: milling time of 2 h, the rotation speed of 500 rpm, and the medium to material weight ratio of 30:1. It should be noted that the media was composed of zirconia beads of different sizes and proportions. The balls with a diameter of 1 cm accounted for 20% of the total mass of the beads, whereas the balls with diameters of 0.6 cm and 0.2 cm accounted for 50 and 30% of the total mass, respectively. All ball-milling treatments were performed in triplicate, and the obtained sample was marked as ball milling bone meal (BBM).

#### Dynamic High-Pressure Microfluidization Treatment

A chicken bone suspension (4% w/w) was prepared by adding CBM to DI water while it was being stirred for 2 min at 10,000 rpm using a high-speed disperser (XHF-D, Ningbo Xinzhi Biological Polytron Technologies Inc., Zhejiang, China). Dynamic high-pressure microfluidization treatment was performed according to the method described by García-Márquez et al. (16) with some modifications based on previous optimization work. The microfluidization step was carried out eight times at 80 MPa using a laboratory-scale microfluidizer (Genizer30k, Suzhou Will NanoBioTech Co., Ltd., Suzhou, China) equipped with an auxiliary processing module of 100 μm H10Z diamond interaction chamber. During this process, the suspension was circulated between the interaction chamber and the cooling coil to cool down immediately after microfluidization. The obtained sample was vacuum freeze-dried using a freeze-dryer (Alpha 1-4 LD plus, Martin Christ Co., Ltd., Osterode, Germany) for 48 h. The sample labeled as dynamic high-pressure microfluidization bone meal (DBM) was stored in a desiccator for further characterization.

### Determination of the Particle Size

The mean particle size of chicken bone was assayed by the methods of Yin et al. (17) with minor modification. Before analysis, the bone powder was ultrasonically dispersed in a 0.2% sodium hexametaphosphate solution for 15 min, forming a 1 mg/g suspension. The particle size distribution in the nanometer range (10–1,000 nm) was determined by dynamic light scattering (DLS) using an analyzer (Nano ZS90, Malvern Instruments Ltd., Worcestershire, United Kingdom) with software Zetasizer 7.02 for data analysis. The particle size of bone in the micrometer range (1–1,000 μm) was determined by a laser particle size instrument (HELOS-OASIS, SYMPATEC Instrument Co., Ltd., Germany). All measurements were performed at 25°C in triplicate.

### Microstructure Observation of Chicken Bone Particles

The morphology of bone particles was observed using a scanning electron microscope (S-3400N and SU70, Hitachi Ltd., Tokyo, Japan) at acceleration voltages of 10 and 5 kV. Prior to observation, three groups of dried bone powders (CBM, BBM, and DBM) were diluted to 1 mg/g with 100% ethanol under an ultrasonic treatment at 100 W for 30 min to avoid serious particle aggregations. Then, 50 μL of diluent was placed onto a clean silicon wafer, dried for 2 h at ambient temperature, and sputter-coated with gold. Microstructure observations were made at a magnification of 5 and 50k for samples, respectively.

### Determination of Zeta Potential

The zeta potential was determined using laser Doppler electrophoresis (LDE) and an analyzer (Nano ZS90, Malvern Instruments Ltd., Worcestershire, United Kingdom), as described by Huang et al. (18) with slight modifications. Prior to analysis, CBM, BBM, and DBM were dispersed in DI water to obtain three suspensions with a solid content of 2 mg/g, after which the pH was adjusted to 7.0 using 0.1 mol/L HCl or NaOH. All measurements were performed by injecting the aforementioned suspension into a folded capillary cell (DTS1070, Malvern Instrument) at 25°C in triplicate.

### Assay of Elements

The contents of elements were measured using an inductively coupled plasma-atomic emission spectrometer (2100DV, Perkin Elmer Co., Ltd., Waltham, MA, United States) in accordance with the method described by Bingöl et al. (19). The samples were pretreated with a microwave digestion apparatus (Mars 6, CEM Co., Matthews, NC, United States). Then, 0.2 g of the sample was taken into a covered polytetrafluoroethylene container, followed by the addition of 7 ml of concentrated HNO_3._ The container was vigorously shaken and placed in a fume hood for 3 h until homogenizing. The homogenate was heated for 30 min following a one-stage digestion program. After cooling and eliminating extra acid, the sample was diluted to 50 ml with DI water and retained as a stock sample solution for element determination.

### X-Ray Diffraction

The X-ray diffraction (XRD) analysis was conducted using a diffractometer (D8 Advance, Bruker Technology Co., Ltd., Rheinstetten, Germany) with Cu-Kα radiation to investigate the phase composition and crystal structure of the bone powders. The diffractometer was run at 40 kV and 30 mA, and the resulting patterns were collected in the 2θ range of 20°–80° at a scanning speed of 2°/min.

### Fourier-Transform Infrared Spectroscopy Spectroscopy

All fourier-transform infrared spectroscopy (FTIR) tests were conducted on an FTIR spectrometer (FT/IR-4700, JASCO Inc., Tokyo, Japan) in the spectral range of 400–4,000 cm^–1^. A mixture of the sample and KBr powder was pressed into pellets. The spectra were transformed against a KBr background.

### Thermogravimetric Analysis

The carbonate contents and thermal behaviors of three samples were determined with a thermal analyzer (STA 2500 Regulus, Netzsch Geraetebau GmbH, Selb, Germany) following a method described by Barinov et al. (20). In the temperature range of 20 to 1,100°C under an airflow, the sample was placed in an Al_2_O_3_ crucible and heated at a constant rate of 10 K/min.

### X-Ray Photoelectron Spectroscopy

The X-ray photoelectron spectroscopy (XPS) measurements were performed following a method described by Maachou et al. (21), using an X-ray photoelectron spectrometer (K-Alpha+, Thermo Fisher Scientific Co., Ltd., Waltham, MA, United States) equipped with a monochromatized aluminum X-ray source. A survey spectrum (0–1,400 eV) was recorded, and high-resolution spectra were obtained for the C 1s, Ca 2p, and P 2p bands. The data were analyzed by the Thermo Avantage v5.9921 software (Thermo Fisher Scientific Co., Ltd., Waltham, MA, United States).

### Calcium Release From Bone Particles

Since the gastrointestinal digestion of chicken bone occurs at pH values of 2 and 7, solutions with pH 2 and 7 were selected to investigate the calcium release in order to mimic the digestion environment. As a pH value between 2 and 7, pH 5 was chosen as well for comparison. According to the methods described by Huang et al. (18), the concentration of calcium ion (Ca^2+^) from the samples was obtained using inductively coupled plasma optical emission spectrometry (ICP-OES) with an ICP apparatus (2100DV, Perkin Elmer Co., Ltd., Hopkinton, MA, United States). Bone powder samples and DI water were mixed and homogenized to obtain a 10 mg/g suspension, of which the pH was subsequently adjusted to 2, 5, and 7 with 0.1 mol/L HCl or NaOH. The mixture was suspended at room temperature for 3 h before being centrifuged at 4,000 *g* for 25 min (5804R, Eppendorf AG, Hamburg, Germany). The supernatant was filtered (0.45 μm, water phase) and diluted with DI water before the test.

### Statistical Analysis

Analysis of variance was analyzed using SPSS Statistics 22.0 (IBM, Armonk, NY, United States). Differences among the means were compared with the Duncan multiple range test at *P* < 0.05.

## Results and Discussion

### Characterization of Particle Size Distribution and Morphology

**FIGURE 1:**
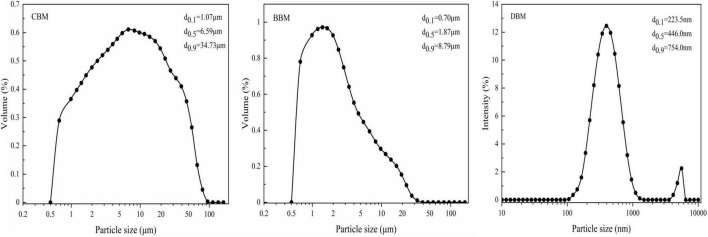
Particle size distributions of chicken bone particles.

The median particle size (D_50_) and particle size distribution (PSD) of chicken bone particles under three treatments are shown in Figure 1. The median particle size of DBM was the smallest (0.45 μm), while that of CBM (6.59 μm) was larger than BBM (1.87 μm). The particle size of CBM is distributed in a wide range, with approximately 79.89% of particles measuring from 0.9 to 30 μm and about 7.37 and 12.74% measuring from 0.5–0.9 to 30–100 μm, respectively. It was notable that ball milling reduced the particle size and narrowed the PSD, as the particle sizes of BBM in the range of 0.5 to 5 μm accounted for 80.36% of the total, and the particle sizes in the range of 5–36 μm accounted for 19.64%. Among all groups, DBM exhibited the smallest particle size and the narrowest PSD, both centered at 0.1–1 μm. More than 90% of the particles in DBM had diameters between 100 and 754 nm, with the large particles near 5 μm due to particle aggregation. Nasiri-Tabrizi et al. (22) observed similar aggregation in hydroxyapatite nanostructures prepared from bovine bones. This may be caused by the extremely high surface energy between the nanoscaled particles.

**FIGURE 2:**
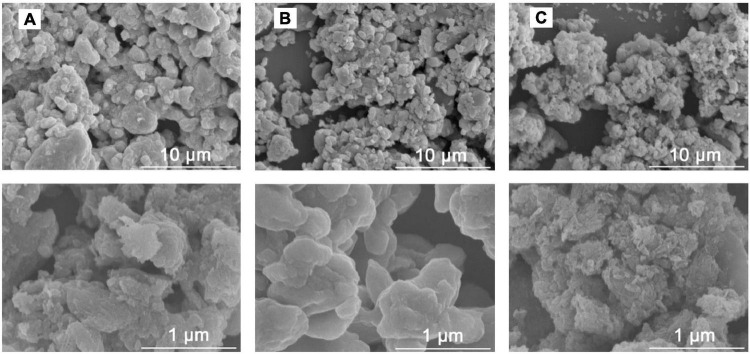
SEM images of the chicken bone particles: **(A)** CBM, **(B)** BBM, **(C)** DBM. The upper and lower lines in the same column indicate the magnifications of 5 and 50k, respectively. CBM, control bone meal; BBM, ball milling bone meal, DBM, DHPM bone meal.

Figure 2 shows the microstructures of CBM, BBM, and DBM samples, respectively. All powders have a certain degree of stacking in general, which may be related to the protein agglomerates adhered to the surface of chicken bones. As seen in Figure 2A, the CBM particles have an irregular morphology composed of heterogeneous polygons, whereas the particles of BBM are more uniform and approximately spheroidal-like. The average particle sizes of BBM and CBM were estimated to be approximately 1 and 5 μm, respectively, as shown in Figures 2A,B, indicating that ball-milling reduced the particle size of bone particles, which is consistent with the results obtained using laser light scattering (Figure 1). Similarly, silver carp bone powder showed a narrower PSD range after ball milling, and the average particle size significantly reduced from 21.75 to 1.75–6.95 μm depending on milling time (23). Unlike the other two samples, the particles of DBM were observed to be loose, spheroidal-like clusters with rough surfaces, increasing specific surface area and blurring the particle boundaries in the field of view.

### Zeta Potential Analysis

**FIGURE 3:**
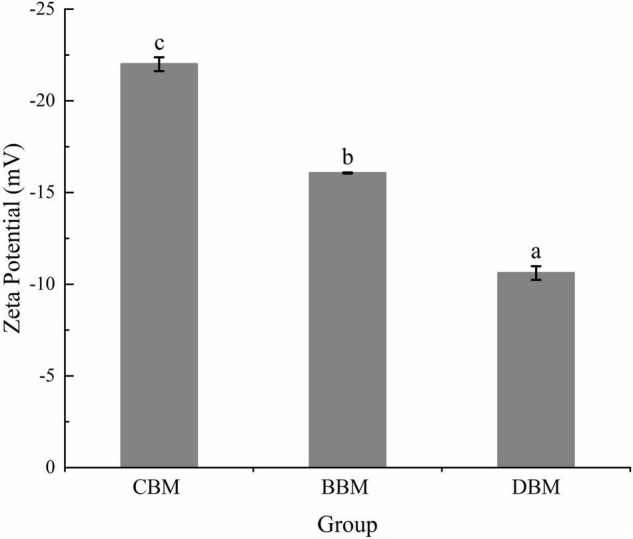
Zeta potential of the chicken bone particle with different treatments. Different lowercase letters indicate significant differences (*p* < 0.05).

The suspension with zeta potential values outside the range of ±30 mV is generally considered stable to the coalescence and flocculation, while those with zeta potential between ±10 and ±30 mV are of incipient instability (24). Zeta potentials of the chicken bone suspensions measured using the DLS method are shown in Figure 3. CBM displayed a zeta potential value of -22.0 mV, whereas the zeta potential values of BBM and DBM samples were -16.1 and -10.6 mV, respectively. The results revealed that even though all three groups of particles were incipiently unstable, CBM particles may have relatively superior stability compared to the other two particles. The results were in accordance with Huang et al. (18), who found that the smaller the particle diameter, the lower the absolute value of zeta potential.

On the other hand, the negative zeta potential of samples in Figure 3 indicated that the surface of bone particles was negatively charged due to the selective adsorption of ions in a colloidal system (25). Zeta potential can be affected by ions’ adsorption to neutralize charges and reduce zeta potential if the ions are opposite to the particle surface charge; otherwise, repelled ions confer electrostatic stabilization (26). Therefore, it can be assumed that part of the calcium ions, which are released from bone particles during milling, reduced the absolute value of zeta potential by neutralizing the negative charge on the particle surface.

### X-Ray Diffraction Analysis

**FIGURE 4:**
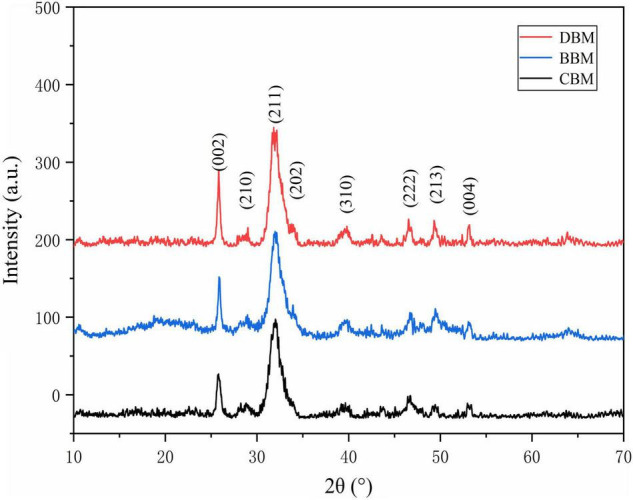
The X-ray diffraction (XRD) patterns of CBM, BBM, and DBM. CBM, control bone meal; BBM, ball milling bone meal; DBM, DHPM bone meal.

The XRD patterns of the CBM, BBM, and DBM samples are shown in Figure 4. The similar diffraction patterns of the three samples confirmed that all of them have a single-phase hydroxyapatite (HAp) structure with a hexagonal crystalline system (JCPDS Card No. 09–0432) despite their different surface morphologies (27).

As shown in the diffraction patterns of the samples, the broad characteristic peak with the highest intensity was observed at a 2θ angle of 31.773°, corresponding to the Miller indices of (211) (28). The diffraction peaks of (112) and (300) related to 32.196 and 32.902° were not separated, indicating a low crystallinity and small crystallite size of HAp in three groups of chicken bone particles (29). The appearance of the rough HAp spectra might be attributed to the incomplete removal of organic portions present in the samples (30), as well as the existence of carbonate ion substitution (31). Carbonate is the most abundant substitution in bone minerals, with 2.3–8 wt% content (32).

Furthermore, the content of the carbonate ions and their positions in the structure, to a large extent, have been known to affect the unit cell parameters (33). As shown in Table 1, the unit cell parameter values of *a* and ***c*** for HAp has been reported to be 9.421 and 6.880 Å, respectively. Precisely, the insertion of CO_3_^2–^ ions into the hexagonal channel of the apatite structure (A-type CHAp) promotes the increase of the parameter and *a* small decrease of the parameter ***c*** in comparison with HAp (34), resulting in the parameter values of 9.527 and 6.875 Å. Conversely, as CO_3_^2–^ partially replaces PO_4_^3–^ ions (B-type CHAp), the lattice parameter *a* decreases (9.386 Å) while ***c*** increases (6.901) (35, 36). The XRD spectra of three samples were structurally refined, and the obtained unit cell parameters were compared with the literature data for HAp, A-type, and B-type carbonated hydroxyapatite (CHAp) (27), as summarized in Table 1. In comparison with the reported HAp, the unit cell parameters of HAp in CBM, BBM, and DBM samples apparently experienced slight variations, which could be related to the substitution of different types of carbonates. It was evident that the parameter values of three samples were inconsistent with those of HAp, A-type, and B-type CHAp, suggesting the coexistence of the three types of apatite. Therefore, it could be concluded that all samples exhibited two types of carbonates for substitution.

**TABLE 1:** The unit cell parameters of CBM, BBM, DBM, HAp, and carbonated (A-and B-types) CHAp.

	CBM	BBM	DBM	HAp	A-type CHAp	B-type CHAp
Space group	P6_3_/m	P6_3_/m	P6_3_/m	P6_3_/m	Pb	P6_3_/m
a(Å)	9.397	9.476	9.416	9.421	9.527	9.386
b(Å)	–	–	–	–	–	–
c(Å)	6.875	6.899	6.892	6.880	6.875	6.901

*“–” means that the value of the same sample is equal to a(Å). The values of HAp, A-type and B-type CHAp were derived from references.*

Based on the above analysis of XRD patterns and unit cell parameters, although the three samples were processed in different methods, they confirmed a resemblance in the phase composition and crystal structure, including low crystallinity, small crystallites, and a certain number of carbonates. FT-IR spectroscopy was carried out to further investigate the two types of cHAp in detail.

### FT-IR Analysis

**FIGURE 5:**
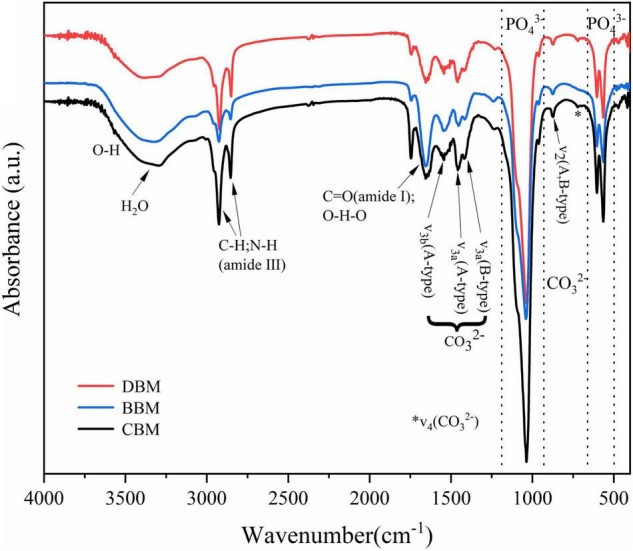
FT-IR spectra of the chicken bone particles with different treatments.

The infrared spectra of the three samples in Figure 5 were similar to those observed in apatite minerals (37), supporting the results observed by XRD. The characteristic bands for the PO_4_^–3^ group consisting of two main regions were illustrated in the spectrum. In the first region, the peak at 962 cm^–1^ corresponded to the *v*1 stretching mode, while the one at 1,034 cm^–1^ was related to the *v*3 stretching mode. The second region of phosphate ions possessed two bands at 603 and 564 cm^–1^, which were attributed to *v*4 bending vibration (38). For carbonate ions, there are usually three vibration bands present in the infrared spectra of carbonate apatite and bone. As shown in Figure 5, all three samples exhibited two notable bands ascribed to carbonate ions, namely the peak near 874 cm^–1^ and the peaks from 1,400 to 1,600 cm^–1^, which belong to the *v*2 out-of-plane bend vibration and the *v*3 asymmetric stretch vibration, respectively. The last peak of carbonate ions usually appeared at 720 cm^–1^, corresponding to the *v*4 band, exhibiting a relatively low intensity that is rarely observed (39). The *v*4 band was weak in CBM and DBM, and was not observed at all in BBM.

The vibrational band of water associated with bone meal HAp samples occurred in the spectral region of 3,000–3,650 cm^–1^ and near 1,650 cm^–1^ (40). A weak shoulder characteristic of hydroxyl stretching vibration might be near 3,570 cm^–1^, which was mostly masked by the fairly broad H_2_O absorptions. The band at 670 cm^–1^ attributed to hydroxyl liberation was too weak to be detected for all samples. Wang et al. (41) reported that the intensity of the band attributed to hydroxyl liberation weakened and almost disappeared with the increase of the carbonate level. Hence, the aforementioned phenomenon could be explained by carbonate substitution.

The band at 1,650 cm^–1^ assigned to the bending C = O (amide I) (42) and the sharp bands appearing at 2,800–3,000 cm^–1^ were recognized as the aliphatic C–H and N–H stretching vibrations (amide III), which revealed the existence of decomposed collagen (the main organic component of bone). Compared with the other two samples, BBM had weaker peaks of the amide III but stronger peaks of the amide I, which might be due to the exposure of more secondary structures caused by the severe destruction of the triple helix structure of the protein after ball milling (43).

**FIGURE 6:**
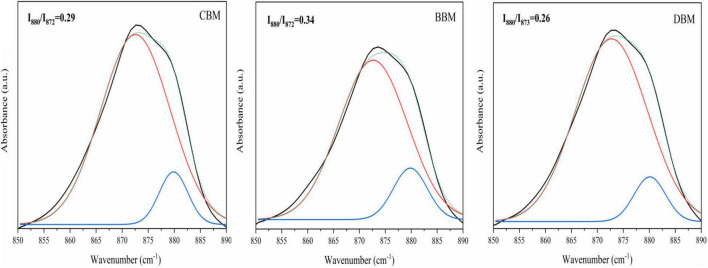
Recorded and fitted 860–890 cm^–1^ FT-IR spectral region of CBM, BBM, and DBM. CBM, control bone meal; BBM, ball milling bone meal; DBM, DHPM bone meal.

Figure 6 displays the spectral region of 850–890 cm^–1^ extracted from the spectra in Figure 5. As shown in Figure 6, the spectra were deconvoluted and fitted by two bands, one for A-type carbonate at 880 cm^–1^ and the other for B-type carbonate at 872 cm^–1^ (35, 44). The resulting peak height ratio of the A- and B-type carbonates in BBM was 0.34, which was in agreement with the 0.33 ratio determined by Markovic et al. (27) in biological hydroxyapatite (BHAp). However, the calculated carbonate peak height ratios in CBM and DBM were 0.29 and 0.26, respectively, which suggested less A-type carbonate substitution in comparison with BBM.

### Concentration of Released Calcium Ions

The XRD analysis showed that the calcium compounds in the chicken bone exist mainly in the form of Hap (Figure 4), which possessed a low solubility. The release of Ca^2+^ at three pHs was assessed using ICP-OES, and the concentrations are summarized in Table 2. Calcium ion concentrations in CBM (control group) at pH 7, 5, and 2 were 16.50, 380.25, and 2364.0 mg/L, respectively. The Ca^2+^ concentration in the suspension increased as the pH value decreased (*P* < 0.05). Additionally, the other two experimental groups demonstrated the same trend, possibly due to the presence of a large amount of H^+^ in an acidic environment (45). The processes of the dissolution of calcium from HAP could be expressed as follows:


a(PO)45OH3⟺4⁢H+2Ca+2+3CaHPO⟺3⁢H+45Ca+2+3HPO2-4

**TABLE 2:** Concentration of Ca^2+^ released from chicken bones in different treatment groups after 3 h in various solvent (mg/L, *n* = 3).

Group	pH = 2	pH = 5	pH = 7
CBM	2364.0 ± 27.622^bA^	380.25 ± 4.693^bB^	16.50 ± 0.015^cC^
BBM	2209.1 ± 25.191^cA^	385.42 ± 7.929^bB^	19.87 ± 0.636^bC^
DBM	2590.6 ± 58.386^aA^	410.83 ± 2.073^aB^	44.50 ± 0.505^aC^

*CBM, control bone meals; BBM, ball milling bone meals, DBM, DHPM bone meals. Different lowercase letters in the same column indicate significant differences between chicken bone meal particles in different treatment groups (p < 0.05); different capital letters in the same row indicate significant differences at different pHs.*

It was notable that the concentration of released calcium ions in DBM at pH 7, 5 and 2 were 44.50, 410.83 and 2590.6 mg/L, higher than that in the other two samples. Particularly, when pH was 7, the calcium ion concentration of DBM (44.50 mg/L) far exceeded that of the other two samples (16.5 mg/L, 19.87 mg/L) by about 2–3 times. This result was consistent with the result of zeta potential. Since small particle size evidently influences the dissolution and release of the effective ingredients through its large specific surface area (23), more release of Ca^2+^ in DBM can be partly attributed to its small size (Figure 1). The Ca^2+^ concentrations in the suspensions could be influenced by both pH and particle size. On the other hand, Zhang et al. (46) prepared fishbone particles *via* wet ball milling after pretreatment at temperatures ranging from 55 to 130°C and found a remarkable difference in enhancing calcium release despite similar particle size, thus illustrating the relationship between calcium release and heating pretreatment and bone breakage efficiencies.

### Thermogravimetric Analysis

**FIGURE 7:**
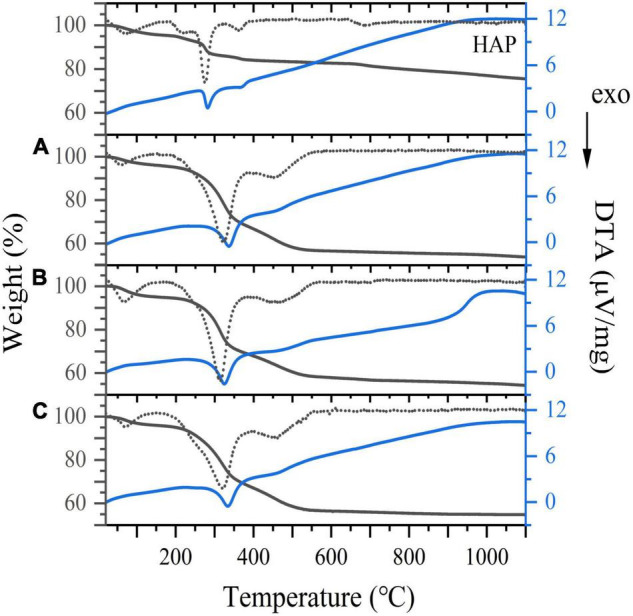
TG/DTA curves for **(A)** CBM, **(B)** BBM, **(C)** DBM and HAP. Dotted line represents TG derivative curve. CBM, control bone meal; BBM, ball milling bone meal; DBM, DHPM bone meal.

The TG/DTA curves of the three samples ranging in temperatures from 20 to 1,100°C are presented in Figure 7. The weight loss stages of three samples at a low temperature were attributed to the loss of surface water at a temperature below 170°C and the removal of lattice water at 380°C (47). When the temperature was increased from 380 to 580°C, the weight loss of the HAp blank group was minimal. In contrast, the mass loss of the three samples was still remarkable, presumably due to the combustion of residual bone organic components (mainly collagen). The third slope between 580 and 950°C was determined to correspond to the evaporation of carbonate ions incorporated in the crystal lattice of bone meal (48). It has been recognized that dehydroxylation does not occur until around 1,000°C (49), hence the mass loss above this point was mainly due to the phase transformation from HAp to tricalcium phosphate. As shown in Figure 7, the estimated amounts of weight loss in the range of 580 to 950°C are about 1.67, 2.46 and 1.86 wt.% for CBM, BBM and DBM, ascribing to the amounts of carbonates in three bone powders. The carbonate content of three samples was lower than the reported data of biological apatite, where the carbonate contents were between 2.3 and 8 wt.% (50). This discrepancy may be related to the congenital conditions of the chicken skeleton raw materials and the treating process (32).

### Elemental Analysis

The mineral comparison of the chicken bone powders with different treatments is collected in Table 3. It was notable that the chicken bone possess certain amounts of Na, K and Mg besides calcium and phosphorus. Venkatesan et al. (37) reported that bone salts are mainly composed of hydroxyapatite (HAp) and calcium hydrogen phosphate, with additional metal ions adsorbed on the surface of these two salts. As the two major mineral components, the contents of calcium and phosphorus were shown in Table 3. The ratio of calcium to phosphorus in CBM, BBM and DBM were 2.18, 1.97, and 2.17, respectively, which was in correspondence with the reported value in the literature (51). The elemental compositions of the three groups showed a significant difference (*p* < 0.05). Among the three groups, the BBM had the highest sodium and potassium content, while the DBM contained the highest content of calcium and phosphate.

**TABLE 3:** Element content of chicken bone power in three different treatment groups.

Elemental composition determined by ICP-OES (mg/g, *n* = 3) Concentration ± SD						
Sample	Ca	P	Na	K	Mg	Ca/P
CBM	217.786 ± 0.992^b^	100.042 ± 0.450^c^	1.603 ± 0.038^c^	0.735 ± 0.017^b^	4.058 ± 0.025^b^	2.18
BBM	204.167 ± 3.399^c^	103.574 ± 0.131^b^	4.634 ± 0.063^a^	11.573 ± 0.139^a^	3.975 ± 0.022^c^	1.97
DBM	234.803 ± 0.405^a^	108.245 ± 0.124^a^	2.358 ± 0.018^b^	0.898 ± 0.000^b^	4.444 ± 0.009^a^	2.17

*CBM, control bone meals; BBM, ball milling bone meals, DBM, DHPM bone meals. Data are expressed as the mean ± SD from triplicate determinations. Different letters in the same column indicate significant differences (p < 0.05).*

### Changes in the Surface Structure of Three Samples

**FIGURE 8:**
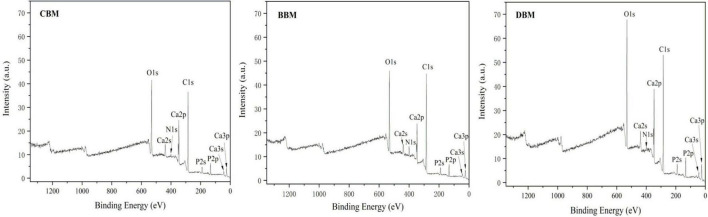
X-ray photoelectron spectroscopy (XPS) survey spectra of CBM, BBM, and DBM. CBM, control bone meal; BBM, ball milling bone meal; DBM, DHPM bone meal.

Figure 8 shows the typical XPS survey spectra of three samples. The characteristic peaks of Ca and P were fitted and analyzed. Through peak area calculation, the surface Ca and P atomic ratios of CBM, BBM, and DBM were 1.25, 1.22, and 1.18, respectively, which were lower than the bulk Ca/P ratio of the three samples in Table 3 (21). Previous studies have reported that calcium salts in bones mainly exist in the form of crystalline HAp, CHAp, and amorphous CaHPO_4_. Therefore, the decrease in the Ca/P ratio could be explained by the fact that the surface calcium composition changed after different treatments, particularly the rise in the content of amorphous CaHPO_4_, leading to a decrease in Ca/P. Thus, it could be assumed that the content of amorphous CaHPO_4_ followed the order of DBM > BBM > CBM, as confirmed by the results of calcium release under different pH values. Since amorphous CaHPO_4_ had better solubility than HAp and was easily dissolved to form calcium ions, dynamic high-pressure microfluidization treatment likely contributed to increased calcium release and hence bioavailability.

## Conclusion

This work investigated the effects of three kinds of typical milling techniques including colloid milling, ball milling, and the dynamic high-pressure microfluidization treatments, on the particle size, microstructure, chemical composition, and calcium release of chicken bone to enhance the utilization efficiency and the added value throughout the chicken meat production chain. The results suggested that the selected milling treatments significantly affected the size, surface structure, and composition of bone particles, affecting the release of calcium ions. Among the three methods, dynamic high-pressure microfluidization treatment showed obvious capability to promote the efficiency of Ca^2+^ release by changing the particle size and surface bone calcium composition, which could be a promising pretreatment technology for use in the food industry for the design and development of potential calcium supplements. However, to maximize the positive effect of microfluidization on natural bone calcium, the correlation between processing parameters and the physicochemical properties of animal bones, particularly the calcium release kinetics, remains to be further studied in future research.

## Data Availability Statement

The original contributions presented in the study are included in the article/supplementary material, further inquiries can be directed to the corresponding author.

## Author Contributions

YW and TF performed the experiments and analyzed the data. YW wrote the original draft. TF and QX contributed to the data curation, writing, and editing. CZ contributed to the formal analysis and editing of the manuscript. YW and JC were responsible for the conceptualization and methodology development. All authors contributed to the manuscript and approved the submitted version.

## Conflict of Interest

The authors declare that the research was conducted in the absence of any commercial or financial relationships that could be construed as a potential conflict of interest.

## Publisher’s Note

All claims expressed in this article are solely those of the authors and do not necessarily represent those of their affiliated organizations, or those of the publisher, the editors and the reviewers. Any product that may be evaluated in this article, or claim that may be made by its manufacturer, is not guaranteed or endorsed by the publisher.
